# Vanillic Acid Restores Coenzyme Q Biosynthesis and ATP Production in Human Cells Lacking *COQ6*

**DOI:** 10.1155/2019/3904905

**Published:** 2019-07-10

**Authors:** Manuel J. Acosta Lopez, Eva Trevisson, Marcella Canton, Luis Vazquez-Fonseca, Valeria Morbidoni, Elisa Baschiera, Chiara Frasson, Ludovic Pelosi, Bérengère Rascalou, Maria Andrea Desbats, María Alcázar-Fabra, José Julián Ríos, Alicia Sánchez-García, Giuseppe Basso, Placido Navas, Fabien Pierrel, Gloria Brea-Calvo, Leonardo Salviati

**Affiliations:** ^1^Clinical Genetics Unit, Department of Women's and Children's Health, University of Padova, Italy; ^2^Istituto di Ricerca Pediatrica (IRP) Città della Speranza, Padova, Italy; ^3^Department of Biomedical Sciences, University of Padova, Italy; ^4^Univ. Grenoble Alpes, CNRS, Grenoble INP, TIMC-IMAG, Grenoble, France; ^5^Centro Andaluz de Biología del Desarrollo, Universidad Pablo de Olavide and CIBERER, Sevilla, Spain; ^6^Laboratorio de Espectrometría de Masas, Instituto de la Grasa (CSIC), Universidad Pablo de Olavide, Sevilla, Spain; ^7^Hematology-Oncology Laboratory Department of Women's and Children's Health, University of Padova, Italy

## Abstract

Coenzyme Q (CoQ), a redox-active lipid, is comprised of a quinone group and a polyisoprenoid tail. It is an electron carrier in the mitochondrial respiratory chain, a cofactor of other mitochondrial dehydrogenases, and an essential antioxidant. CoQ requires a large set of enzymes for its biosynthesis; mutations in genes encoding these proteins cause primary CoQ deficiency, a clinically and genetically heterogeneous group of diseases. Patients with CoQ deficiency often respond to oral CoQ_10_ supplementation. Treatment is however problematic because of the low bioavailability of CoQ_10_ and the poor tissue delivery. In recent years, bypass therapy using analogues of the precursor of the aromatic ring of CoQ has been proposed as a promising alternative. We have previously shown using a yeast model that vanillic acid (VA) can bypass mutations of *COQ6*, a monooxygenase required for the hydroxylation of the C5 carbon of the ring. In this work, we have generated a human cell line lacking functional *COQ6* using CRISPR/Cas9 technology. We show that these cells cannot synthesize CoQ and display severe ATP deficiency. Treatment with VA can recover CoQ biosynthesis and ATP production. Moreover, these cells display increased ROS production, which is only partially corrected by exogenous CoQ, while VA restores ROS to normal levels. Furthermore, we show that these cells accumulate 3-decaprenyl-1,4-benzoquinone, suggesting that in mammals, the decarboxylation and C1 hydroxylation reactions occur before or independently of the C5 hydroxylation. Finally, we show that *COQ6* isoform c (transcript NM_182480) does not encode an active enzyme. VA can be produced in the liver by the oxidation of vanillin, a nontoxic compound commonly used as a food additive, and crosses the blood-brain barrier. These characteristics make it a promising compound for the treatment of patients with CoQ deficiency due to *COQ6* mutations.

## 1. Introduction

Coenzyme Q (CoQ) is a key component of the mitochondrial respiratory chain (RC) where it shuttles electrons from complexes I and II to complex III. It is also a cofactor of other mitochondrial dehydrogenases and of uncoupling proteins, an antioxidant, and a modulator of the mitochondrial permeability transition pore [1]. CoQ is comprised of a quinone group and of a polyisoprenoid tail, which varies in length in different species: ten isoprene units in humans (CoQ_10_), nine in mice (CoQ_9_), and six in yeast (CoQ_6_). The CoQ biosynthetic pathway is still incompletely characterized, especially in higher eukaryotes [2]. The biogenesis of the isoprene tail begins in the cytosol through the mevalonate pathway, sharing its initial steps with cholesterol biosynthesis. The individual isopentyl diphosphate units produced in these pathways are then joined together to form the all-trans polyprenyl tail by *COQ1* (in yeast) and *PDSS1* and *PDSS2* (in mammals) within mitochondria. The quinone group is synthesized from tyrosine through a poorly characterized set of reactions that produce 4-hydroxybenzoate (4-HB) [3, 4]. 4-HB is then joined to the polyisoprene tail by COQ2, an integral protein of the mitochondrial inner membrane [5]. Subsequent biosynthetic steps occur within the mitochondrial matrix and are catalyzed by a set of enzymes that modify the quinone ring. In eukaryotes, these enzymes form a multiprotein complex associated with the inner mitochondrial membrane [6]. This complex is still poorly characterized in mammals, and the precise sequence of reactions is not known [2].

Among the different genes coding the components of this complex, *COQ6* encodes a FAD-dependent monooxygenase responsible for the addition of the hydroxyl group in position C5 of the quinone ring [7].

CoQ biosynthesis is relevant for human diseases because mutations in genes involved in CoQ biosynthesis cause primary CoQ deficiency, a clinically heterogeneous group of disorders. In particular, patients with mutations in *COQ6* present with steroid-resistant nephrotic syndrome (SRNS) associated with sensorineural deafness and a variable degree of encephalopathy [8]. CoQ-deficient patients respond to oral CoQ_10_ supplementation, making this one of the few treatable mitochondrial disorders [9]. Therapy is however still problematic; CoQ_10_ has a low bioavailability and very high doses are required; moreover, not all patients seem to respond adequately to treatment. Long-term follow-up data in humans are not available, but in a mouse model of CoQ deficiency, which recapitulates the human renal phenotype, progression of the disease is observed in the long term, despite treatment [10].

A possible solution to this problem is bypass therapy using analogues of 4-HB, which provide the defective chemical group and can reactivate endogenous CoQ biosynthesis [11, 12]. In the case of *COQ6*, it has been shown in yeast mutants that 4-HB analogues such as vanillic acid (VA) or 3-4-dihydroxybenzoate, which already harbor a hydroxyl or methoxyl group in position 5 of the ring, may bypass the COQ6 defect and restore endogenous biosynthesis [7]. For this to occur, however, the mutants must retain structural stability, in order to allow formation of the multienzyme complex (in yeast, the presence of each of its protein components is required for the stability of the complex). *COQ6* mutations found in human patients have these characteristics, and when expressed in *S*. *cerevisiae*, they are responsive to VA [13], making this compound an attractive alternative for patient treatment.

In this work, we report the generation of a cell line lacking functional *COQ6*, which was used to test the effect of VA supplementation.

## 2. Materials and Methods

### 2.1. Cell Culture and Reagents

HEK 293 cells were cultured at 37°C using DMEM 4.5 g/L glucose, L-glutamine (6 mM) (Gybco™) supplemented with sodium pyruvate solution (1 mM) (Sigma), an antibiotic/antimycotic solution (Sigma), uridine (10 *μ*M) (Sigma), and 10% fetal bovine serum (FBS) (Gybco™). When required, supplemental CoQ prediluted in ethanol 100% was added to the medium at a final concentration of 100 *μ*M (coenzyme Q_10_, ≥98%, HPLC, Sigma) and supplemental vanillic acid was added at a final concentration of 500 *μ*M (vanillic acid, ≥97%, HPLC, Sigma.). For all biochemical assays, cells were incubated for 48 hours in DMEM containing 2 mM glucose and 5% FBS, to force mitochondrial respiration.

### 2.2. Generation of CRISPR Knockout Cell Lines

The CRISPR/Cas9 constructs were purchased from transOMIC technologies. We performed transfection by using Lipofectamine 2000 (Life Technologies), according to the manufacturer's instructions. Two pCLIP-All-EFS vectors, which express Cas9 and the target gRNAs, together with the puromycin resistance gene (TEVH-1164665: 3′-TCCTGTAGAGAACCGTCACT-5′; TEVH-1097523: 3′-CTAGGGTAATATGAACCCAA-5′), were cotransfected into HEK293 cells. Cells were incubated in medium with 1,5 *μ*g/mL puromycin for selection. Cell clones were obtained by cell sorting into 96-well plates, cultured until confluence, and duplicated for genotyping PCR. Culture media were supplemented with CoQ_10_ and uridine as described [14]. Three positive clones were expanded and mixed (1 : 1 : 1) to obtained a homogeneous cell population.

### 2.3. Construction and Transfection of Lentiviral Vectors

The coding sequence of human *COQ6* isoform *a* and isoform *c*, the mutated sequence *COQ6* G255R, and a negative control expression sequence were transferred into the lentiviral expression plasmid (pLenti6/V5-DEST™ Gateway™ Vector, Invitrogen). To generate lentiviral particles, vectors were cotransfected with packaging vectors (ViraPower Packaging Mix, Invitrogen) into HEK293-FT cells using Lipofectamine 2000 (Life Technologies). Culture supernatants were harvested on day 3 and used to transduce HEK293 *COQ6KO* cells. Selection was carried out as described [15].

### 2.4. GFP Fusion Vectors

A *COQ6*_Iso_*c*-GFP fusion gene was constructed using the pEGFP-N1 as previously reported [8], except that the forward primer employed was specific for *COQ6* isoform *c* (5′-TCTAAGCTTGCTATGCGGGGCCAGGGTCCACC-3′). As the PCR template, we employed the plasmid generated for the lentiviral construct. The pEGFPN1-COQ6_Iso_*c* was used to transiently transfect HeLa cells stably expressing mtRFP seeded on glass coverslips [16]. 48 hours after transfection, cells were examined using a Zeiss Axio Imager M2 fluorescence microscope.

### 2.5. Immunoblot Assay

Standard techniques were employed for SDS-PAGE and Western blotting to PVDF membranes using the antibody COQ6 12481-1-AP (Proteintech). Peroxidase-conjugated anti-rabbit IgG was used as secondary antibodies (Santa Cruz). Visualization of antibody protein complexes was achieved by enhanced chemiluminescence (LiteAblot Turbo, EuroClone) and the ChemiDoc™ XRS+ System (Bio-Rad).

### 2.6. Lipid Extraction and HPLC Analysis

Harvested cells (1 mg protein) were resuspended in 0.3 mL of a 0.15 M KCl solution. Then, 200 *μ*L glass beads, 10 *μ*L of a 5 *μ*M UQ8 standard solution, and 3 mL methanol were added. The tubes were vortexed for 1 min, 2 mL petroleum ether (40-60° boiling range) was added, and vortex was repeated for 1 min. The tubes were centrifuged at 700 rpm for 1 min, the upper phase was collected, and the methanol phase was extracted again with 2 mL petroleum ether. Both petroleum ether phases were combined and dried under a nitrogen flow, and the lipid extracts were resuspended in 200 *μ*L ethanol. HPLC analysis was conducted essentially as described [17] with the following modifications. Samples corresponding to 0.2 mg protein were injected onto the C18 column, and separation was obtained at a flow rate of 1 mL/min with a mobile phase composed of 25% isopropanol, 45% methanol, 20% ethanol, and 10% of a solution composed of 90% (*v*/*v*) isopropanol, 10% (*v*/*v*) 1 M ammonium acetate, and 0.1% (*v*/*v*) formic acid. The precolumn electrode (5020 Guard Cell, Thermo) was set either at +650 mV (oxidizing mode) or at -650 mV (reducing mode). Mass spectrometry detection was conducted in positive mode with electrospray ionization, probe temperature of 400°C, and cone voltage of 80 V. 4-HP_10_ was detected with single-ion monitoring at *m*/*z* 806.5 (M + NH_4_^+^) and its reduced form, 3-decaprenyl-1,4-benzoquinol, at *m*/*z* 808.5 (M + NH_4_^+^).

CoQ_10_ biosynthesis rates were measured as previously described [18], by evaluating incorporation of ^14^C-radiolabelled 4-HB.

### 2.7. Respiratory Chain Activities and ATP Levels

Activities of mitochondrial respiratory chain complexes were measured as described [19]. ATP levels in cells were determined using the ATPlite Luminescence Assay System (PerkinElmer) according to the manufacturer's instructions and using a Victor3 (PerkinElmer) multilabel plate reader.

### 2.8. ROS Measurement

Intracellular ROS was measured using two different assays. To measure the mitochondrial redox state, we used the mitochondrially targeted redox-sensitive GFP (roGFP) system [20]. A vector expressing ro-GFP was transfected into the different cell lines using Lipofectamine 2000 (Life Technologies). After 48 hours of growth and treatment, fluorescence was measured in a Victor3 (PerkinElmer) multilabel plate reader with an excitation of 405 nm and emission of 485 nm.

Total oxidant levels were measured using the oxidant-sensitive fluorescent probe CM-H2DCFDA (Invitrogen). ROS were detected using CM-H2DCF-DA, a chloromethyl derivative of H2DCFDA. Cells were seeded onto 24 mm diameter glass coverslips placed in 6-well plates and cultured in the appropriate medium as described above. 30 min before measurements, cells were loaded with CM-H2DCF-DA (2.5 *μ*M) and then washed twice. All the steps were carried out at 37°C with 5% CO_2_. The chambered coverslips were transferred to a Leica (Wetzlar, Germany) DMI6000B microscope, equipped with a digital camera. Fluorescence was measured in 5-7 random fields per chamber. For each group, 4-6 chambers were analyzed. Fluorescence emission was monitored by using 560 ± 20 nm excitation and 645 ± 37 nm emission filter setting. Data were acquired and analyzed using MetaFluor software (Universal Imaging).

### 2.9. Measurement of Oxygen Consumption by the Seahorse XF24 Extracellular Flux Analyzer

The oxygen consumption rate (OCR) was determined using a Seahorse XF24 Extracellular Flux Analyzer following the manufacturer's instructions. 24 h before seeding the Seahorse plate, cells were treated with vanillic acid or CoQ. 10^3^ cells per well were seeded onto poly-D-lysine-precoated (Sigma) Seahorse 24-well plates for 48 hrs. Then, medium was replaced for 2 mM glucose DMEM and cells were incubated with this medium for 24 h. Prior to the measurements, medium was replaced with Seahorse XF base medium supplemented with 2 mM glucose, 2 mM glutamine, and 1 mM sodium pyruvate and incubated for 1 hr at 37°C without CO_2_. OCR was measured under basal conditions and after the sequential addition of oligomycin (1 *μ*M), FCCP (0.2 *μ*M), rotenone (1 *μ*M), and antimycin A (2.5 *μ*M). To normalize respiration rates, cells were harvested and counted after the assay.

### 2.10. In Silico and Statistical Analyses

The structure of human COQ6 was modelled on that of *P*. *fluorescens* para-hydroxybenzoate hydroxylase (Protein Data Bank code 1PBE) as reported [13]. Statistical analyses were performed as described [21].

## 3. Results

### 3.1. COQ6 Is Essential for CoQ_10_ Biosynthesis in Mammalian Cells

**Figure 1:**
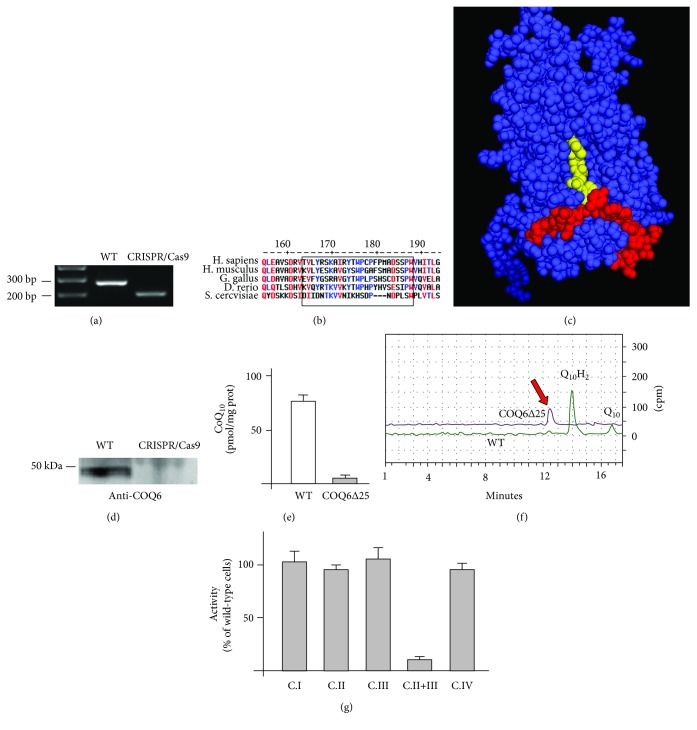
(a) PCR amplification of the *COQ6* genomic region targeted by the CRISPR/Cas9 nucleases showing the 75 bp deletion in CRISPR/Cas9-treated cells. (b) Alignment of COQ6 proteins from different species. Boxed are the 25 amino acids affected by the deletion. The numbers refer to the human protein. (c) Three-dimensional structure of human COQ6. In red are the deleted amino acids, and in yellow are the bound FAD molecules. (d) Western blot analysis in wild-type and COQ6∆25 cells. (e) Steady-state CoQ_10_ levels in wild-type and COQ6∆25 cells. (f) ^14^C 4-HB incorporation in wild-type and COQ6∆25 cells. The peaks corresponding to oxidized and reduced CoQ_10_ are virtually undetectable in COQ6∆25 cells. An additional peak (red arrow) is present in COQ6∆25 cells. (g) Respiratory chain enzyme activities normalized to citrate synthase activity of COQ6∆25 cells. Data are expressed as percentage of activity of control cells.

Wild-type HEK293 cells were transfected with the CRISPR-Cas9 constructs, and after selection, individual clones were genotyped. All analyzed clones harbored a deletion of 75 nucleotides (Figure 1(a)), which caused a deletion of 25 amino acids (Figure 1(b)) in a region of the protein which participates to the FAD-binding site (Figure 1(c)) and is contained in all three isoforms of human *COQ6*.

Western blot analysis of the mixed clones did not detect residual COQ6 protein (Figure 1(d)), and when the mutant cDNA was expressed in delta *coq6* yeast, VA could not rescue growth in nonfermentable media (not shown). These results indicate that the deletion results in an inactive, unstable protein. From now, we will refer to these cells as COQ6∆25.

CoQ_10_ levels were markedly reduced in COQ6∆25 cells compared to wild-type cells (Figure 1(e)). Incorporation of ^14^C-labelled 4-HB was virtually undetectable (Figure 1(f)), indicating that, in analogy with what we observed in *COQ4KO* cells [22], the residual CoQ_10_ detected in these cells is not produced endogenously but it is derived from the medium. The chromatogram showed an additional radioactive peak present only in COQ6∆25 cells, which eluted faster than CoQ_10_ (Figure 1(f)).

Activities of individual respiratory chain enzymes were normal; however, combined activity of complexes II and III was markedly reduced in these cells, consistent with severe CoQ_10_ deficiency (Figure 1(g)). ATP levels were also markedly reduced (see below). Complex I + III activity was not assayed as results are unreliable in cultured (even when mitochondria-enriched preparations are used), due to the residual high levels of rotenone-insensitive activity [19].

### 3.2. COQ6∆25 Cells Accumulate 3-Decaprenyl-1,4-benzoquinone

**Figure 2:**
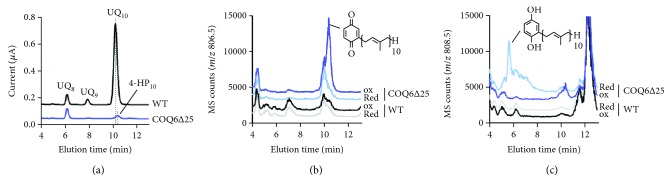
(a) HPLC electrochemical detection analyses (with precolumn electrode in oxidizing mode) of lipid extracts from wild-type (WT) and COQ6∆25 cells (0.2 mg protein) with UQ_8_ used as standard. UQ_10_ and 4-HP_10_ have different retention times as shown by the dotted lines. (b, c) Single-ion monitoring ((b) *m*/*z* 806.5, (c) *m*/*z* 808.5) in HPLC mass spectrometry analyses of lipid extracts from WT and COQ6∆25 cells (0.2 *μ*g protein) with the precolumn electrode set in oxidizing mode (ox) or reducing mode (red). The chemical structures of (b) 3-decaprenyl-1,4-benzoquinone and (c) 3-decaprenyl-1,4-benzoquinol are shown.

To characterize the additional product detected in COQ6*∆*25, we analyzed lipid extracts from WT and COQ6*∆*25 cells by HPLC-MS. Electrochemical detection confirmed a marked reduction of CoQ_10_ in COQ6∆25 cells and the presence of an electroactive compound that eluted at 10.4 min, right after CoQ_10_ (Figure 2(a)). This compound was characterized by HPLC-coupled mass spectrometry, and mass scanning (*m*/*z* 600-900) showed a prominent ion at 806.5 around 10.4 min (data not shown). A signal at 10.4 min was indeed specifically obtained in COQ6∆25 cells with single-ion monitoring for *m*/*z* 806.5 (Figure 2(b)), a mass (M + NH_4_^+^) compatible with that of 3-decaprenyl-1,4-benzoquinone (4-HP_10_). Upon shifting the precolumn electrode to a reducing mode, the signal at *m*/*z* 806.5 disappeared (Figure 2(b)) and a signal at *m*/*z* 808.5 appeared at 5.8 min (Figure 2(c)), consistent with the two-electron reduction of 4-HP_10_. Overall, these data show that the impairment of CoQ_10_ biosynthesis in COQ6∆25 cells leads to the accumulation of 4-HP_10_, in agreement with the accumulation of 4-HP_6_ previously reported in yeast Δcoq6 cells [7].

### 3.3. COQ6 Isoform c Does Not Rescue CoQ_10_ Biosynthesis

**Figure 3:**
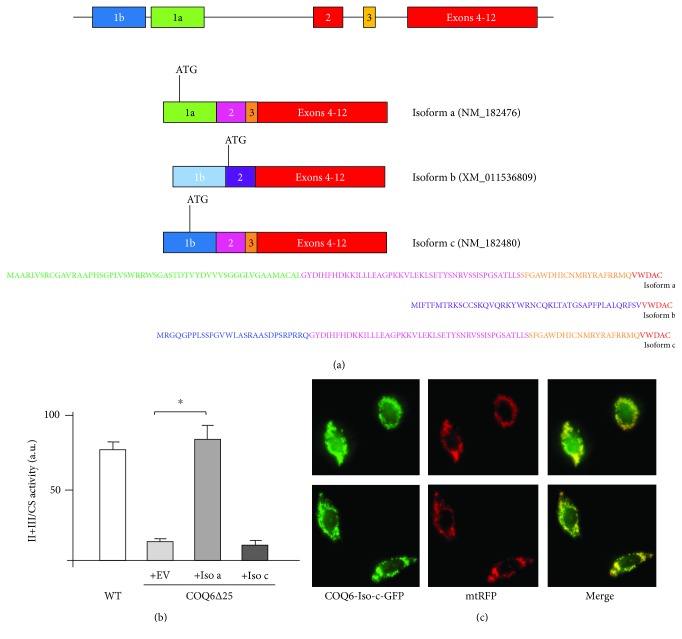
(a) Schematic representation of the three main COQ6 isoforms. Isoform *a* is the most widely represented and comprises exon 1a. Isoform *b* is transcribed from an alternative first exon (exon 1b) which is not translated, and translation starts in exon 2 using a different reading frame compared to those of isoforms *a* and *c*. Exon 3 is skipped and this allows restoration of the normal reading frame from the 3′ exons. Isoform *c* is transcribed and translated from exon 1b. (b) Complex II + III activity in wild-type (WT) and COQ6∆25 cells transduced with the empty vector (EV), *COQ6* isoform *a* (Iso *a*), and *COQ6* isoform *c*. ^∗^*p* < 0.05. (c) HeLa cells stably expressing mtRFP were transiently transfected with COQ6-Iso-*c*-GFP plasmid expressing GFP fused to the C-terminus of COQ6.

At least three different *COQ6* isoforms exist in human cells: the most represented transcript (with GenBank accession NM_182476) encodes isoform *a*, but there are two additional transcripts (isoforms *b* and *c*), which are present at lower levels in cells and encode different proteins [8]. We have previously shown that isoform *b*, which contains an alternative first exon (exon 1b) and lacks exon 3, is not active [13]. Isoform *c* (accession NM_182480) differs from isoform *a* only for the first exon and is still predicted to contain a mitochondrial-targeting sequence, but it is not clear if it encodes an active enzyme (Figure 3(a)). We employed our model to investigate the role of isoform *c*. Using lentiviral vectors, we expressed either isoform *a* or isoform *c* in *COQ6KO* cells. After selection, cells were incubated in DMEM containing 2 mM glucose for 48 hrs. Only isoform *a* rescued complex II and III activity (and thus CoQ production), whereas isoform *c* had no effect (Figure 3(b)). We also checked the subcellular localization of isoform *c* using a C-terminal GFP fusion construct, analogous to what we had previously employed for isoform *a*. As seen in Figure 3(c), the fusion protein colocalizes with mitochondrially targeted red fluorescent protein (mtRFP), indicating that the lack of complementation is not due to faulty mitochondrial targeting. We noted that some diffuse, faint green florescence can be observed also in the cytosol. This could reflect the fact that, physiologically, a certain proportion of COQ6 isoform *c* is not imported into mitochondria but it could also be an artifact due to overexpression or to the presence of GFP.

### 3.4. Vanillic Acid Restores CoQ_10_ Biosynthesis, Cellular Respiration, and ATP Production in COQ6∆25 Cells

**Figure 4:**
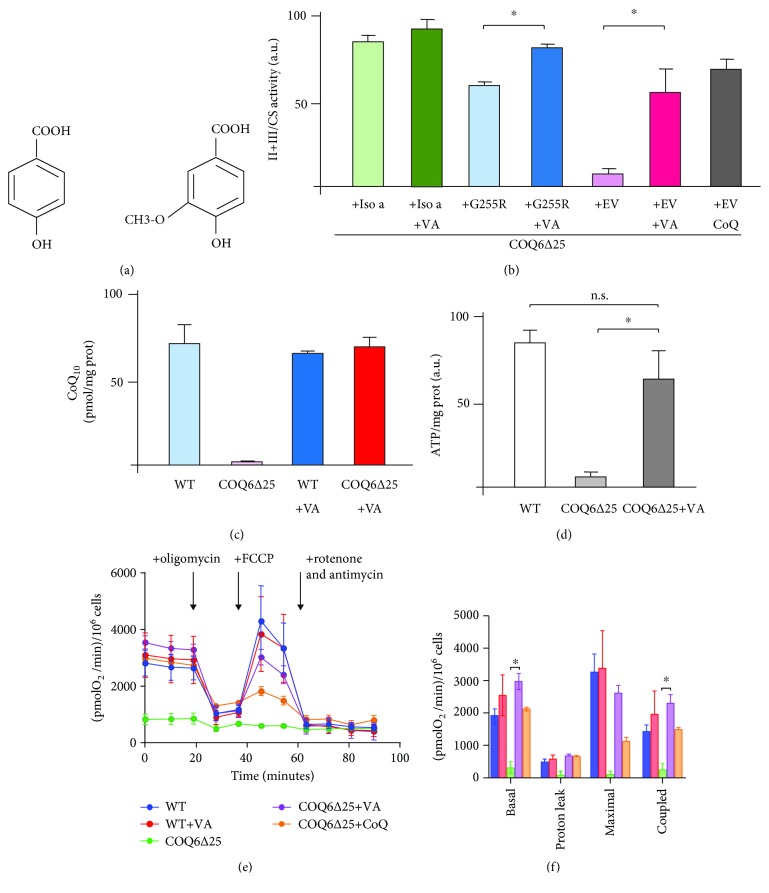
(a) Structure of VA compared to 4-HB the physiological precursor of CoQ. (b) Complex II + III activity in COQ6∆25 cells transduced with *COQ6* isoform *a* (Iso *a*), the G255R mutant, and the empty vector (EV), before and after supplementation with VA or CoQ_10_; n.s.: nonsignificant. (c) Steady-state CoQ10 levels and (d) ATP levels in WT and COQ6∆25 cells after supplementation with VA for 48 hours; a.u.: arbitrary units. (e) Oxygen consumption rate (OCR) profiles in WT and COQ6∆25 cells treated with VA or CoQ, determined using a Seahorse XF24 Extracellular Flux Analyzer. The arrows indicate the addition of the individual inhibitors. (f) The same data represented as histograms.

VA differs from 4-HB for the presence of a methoxyl group in position C5 (Figure 4(a)). To test the possibility of bypassing the COQ6 defect in mammalian cells, we incubated different cell types with either CoQ_10_ or VA. We found that VA restored II + III activity in COQ6*∆*25 cells transduced with the G255R point mutant, but also in cells transformed with the empty vector, indicating that contrary to what happens in yeast, rescue is not restricted to inactive but structurally stable alleles but it occurs also with *null* mutants. CoQ_10_supplementation had a similar effect on II + III activity (Figure 4(b)). Direct measurement of CoQ_10_ levels confirmed this finding (Figure 4(c)). We noted that the G255R mutant displays relatively high basal II + III activity but this is consistent with the fact that it is a hypomorphic allele [13] and that the lentiviral vector that we are using is probably overexpressing the transgene since it uses the strong CMV promoter. ATP levels were markedly reduced in COQ6*∆*25 cells, but after VA supplementation, they were virtually normal (Figure 4(d)). VA treatment could also restore coupled respiration in COQ6*∆*25 cells (Figures 4(e) and 4(f)). In untreated cell, this value was about 10% of the wild type, in line with what we reported for COQ4KO cells. The residual respiration is probably due to the presence of a small amount of exogenous CoQ collected from the serum in cell culture media [22].

### 3.5. VA but Not CoQ10 Normalizes ROS Production in COQ6∆25 Cells

**Figure 5:**
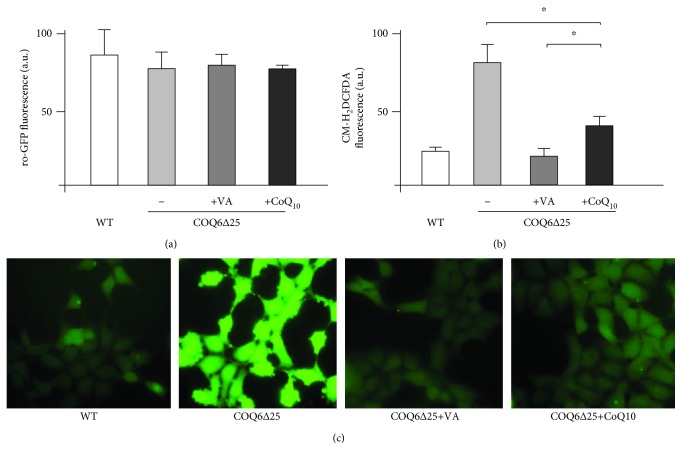
(a) Mitochondrial ROS evaluated using mitochondrially targeted ro-GFP. (b, c) Total cellular ROS evaluated using the CM-H_2_DCFDA probe (see methods for details). (c) Representative photomicrographs of the same cells (magnification ×40).

Because of the role of CoQ as an antioxidant, we studied the effect of VA supplementation on ROS production in COQ6*∆*25 cells. In accordance with previously reported data [23], we did not detect a significant increase of mitochondrial ROS production using mitochondrially targeted ro-GFP (Figure 5(a)). Conversely, when we employed a different system, based on the CM-H_2_DCFDA probe, which measures total cellular ROS [24], we found increased levels in the COQ6*∆*25 cells. VA treatment was able to decrease ROS to basal levels, while CoQ supplementation was only partially effective (Figures 5(b) and 5(c)).

## 4. Discussion

Bypass therapy is a promising alternative to conventional CoQ supplementation for patients with primary CoQ deficiency and has been successfully tested in mouse models of *COQ7* and *COQ9* defects [25, 26] and in cells of patients with *COQ7* mutations [27, 28]. In particular, mice with a conditional ablation of the *Coq7* gene (which encodes the C6-hydroxylase catalyzing the penultimate step of CoQ biosynthesis) at 2 months of age developed severe, multiorgan CoQ deficiency, which lead to death after 9 months. If these animals were treated immediately before death with 2,4-dihydroxybenzoate (2,4-DHB), a compound similar to VA which can bypass the Coq7 defect, CoQ biosynthesis was restored and the animals recovered virtually all symptoms and displayed a normal lifespan [26].

In this work, we have generated a cell line lacking functional *COQ6* using a genome-editing approach. We are aware that this model has some limitations. We obtained an in-frame deletion, which could still produce some folded protein (below the threshold of detectability of our assays), and we could not rule out off-target effects, even though reexpression of the wild-type cDNA corrected the biochemical phenotype of these cells.

Using this model, we showed that VA can restore CoQ_10_ endogenous biosynthesis in human cells lacking *COQ6.* VA is a very attractive compound for use as a therapeutic agent in patients for several reasons. In vivo, it can be produced in the liver through oxidation of vanillin [29, 30], which is commonly used as a flavoring agent by the food industry. Vanillin is considered nontoxic and safe for human use by the FDA and has a good bioavailability. VA can cross the blood brain barrier efficiently [31], thus overcoming one of the major limitations of oral CoQ supplementation.

Moreover, we show that in our cellular model, VA can correct cytosolic ROS production, which is increased in COQ6*∆*25 cells and is only partially reduced by CoQ supplementation, confirming the observation that in *C. elegans* reactivation of endogenous biosynthesis is superior to CoQ supplementation [32]. These data are not in contrast with previous results in patients' cultured fibroblasts [23]. Exogenous CoQ_10_ enters the cells through the brefeldin A-sensitive endo-exocytic pathway, and it is mainly incorporated into the endolysosomal fraction and also in mitochondria-associated membranes and mitochondria. The better performance of VA compared to CoQ could be explained by the fact that VA restores the production of endogenous CoQ, which is then delivered to the appropriate subcellular compartments at optimal concentration.

Our work highlights other important points regarding COQ6 function in mammalian cells. *COQ6* isoform *c* cannot rescue CoQ biosynthesis when expressed in COQ6*∆*25 cells, suggesting that it does not encode an active protein. This finding is critical for the correct interpretation of genomic analyses in patients. In fact, *COQ6* exon 1b is targeted by most exome analysis kits and the gnomAD database (http://gnomad.broadinstitute.org/gene/ENSG00000119723) lists several possible loss-of-function mutations within this exon. One of them NM_182480:c.41G>A p.(Trp14∗) occurs with a minor allelic frequency of 0.05 in Africans and is present also in the homozygous state in apparently normal individuals. Overall, these observations indicate that *COQ6* isoforms *b* and *c* are not essential and are not involved in CoQ deficiency. A regulatory function has been postulated for other inactive isoforms of *COQ* genes [33–35], but to date, no clear proof of these hypotheses has been provided yet.

**Figure 6:**
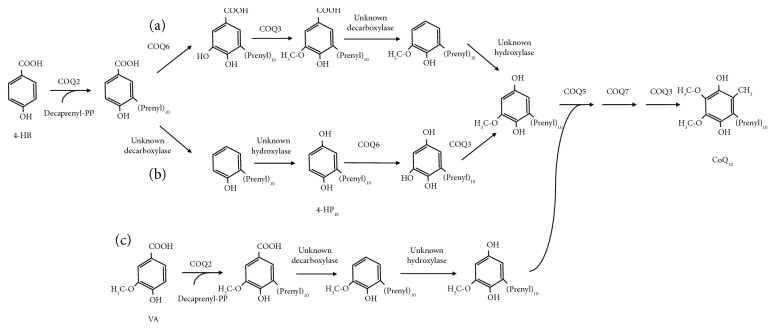
Proposed models of the CoQ_10_ biosynthetic pathway in mammals showing two alternative sequences of reactions (the decaprenyl chain is abbreviated as R). Path (a) corresponds to the traditional model in which COQ6 acts after the condensation of the ring with the isoprene tail, while path (b) shows the unknown decarboxylase and hydroxylase (in blue) acting before COQ6, consistent with the accumulation of 4-HP_10_ in COQ6-deficient cells. Path (c) depicts the same pathway when VA is employed instead of 4-HB. Biosynthesis can occur even in the absence of COQ6 because carbon C5 of the ring is already bound to a methoxyl group in VA.

The exact order of reactions that carry out the modifications of the aromatic ring of CoQ is still unclear in eukaryotes. Generally accepted models indicate that the C5 hydroxylation catalyzed by COQ6 is the first reaction to occur after the condensation of 4-HB with the polyisoprene tail [36]. However, the fact that *COQ6* knockout cells (both human and yeast) accumulate 4-HP, a compound that is decarboxylated and hydroxylated in position C1 of the ring, indicates that these reactions (which are carried out by still unidentified enzymes) occur before or independently on C5 hydroxylation. Furthermore, this result also confirms the notion that C1 hydroxylation is not catalyzed by COQ6 [7]. The identification of the enzymes that catalyze these biosynthetic steps will be critical to validate this hypothesis.  Figure 6 depicts a possible model of CoQ biosynthesis in mammals according to our data.

VA was able to restore biosynthesis even in the presence of a *null* mutant, implying that, contrary to yeast, mammalian cells can assemble the biosynthetic complex even in the absence of COQ6. Therefore, data obtained in yeast about complex assembly and stability should be extrapolated with caution to mammalian cells.

An open issue is whether all *COQ6* mutations could be responsive to VA. In the case of *COQ7*, the response to bypass therapy with 2,4-DHB was more evident when CoQ biosynthesis was severely impaired, rather than when the defect was relatively mild [28]. In the case of COQ6, VA was effective in both situations (CoQ biosynthesis was virtually absent in COQ6*∆*25 as in COQ7KO cells, while the biosynthetic defect in cells expressing the G255R mutant was similar to that found in cells with the mild *COQ7* [M103T+L111P] allele). This peculiar behavior of *COQ7* mutants was attributed to the fact that 2,4-DHB has also an inhibitory action on CoQ biosynthesis [28]. Conversely, VA apparently has no inhibitory effects on the pathway and it is reported to stimulate biosynthesis of other COQ proteins [11]. We have tested the majority of known human mutations in a yeast model, and they all appeared to be responsive to VA [13]. Nevertheless, before attempting to treat patients with VA, one should consider to assay the efficacy of the compound in cell lines (primary fibroblasts or lymphoblastoid cells) obtained directly from the patient.

## Data Availability

The data used to support the findings of this study are available from the corresponding author upon request.
